# Nutritional Quality and Safety Assessment of Pork Meat Cuts from Romania: Fatty Acids and Elemental Profile

**DOI:** 10.3390/foods13050804

**Published:** 2024-03-05

**Authors:** Florina-Dorina Covaciu, Ioana Feher, Gabriela Cristea, Adriana Dehelean

**Affiliations:** National Institute for Research and Development of Isotopic and Molecular Technologies, 67-103 Donat Street, 400293 Cluj-Napoca, Romania; florina.covaciu@itim-cj.ro (F.-D.C.); ioana.feher@itim-cj.ro (I.F.); gabriela.cristea@itim-cj.ro (G.C.)

**Keywords:** fatty acids, pork meat, nutritional indices, toxic and potentially toxic elements, GC-FID, ICP-MS

## Abstract

In this study, the fatty acids and elemental profiles of 53 pork cut samples were determined. To offer insights into their potential health implications, we computed 18 key nutritional indices. These indices included parameters such as saturated fatty acids (SFAs), monounsaturated fatty acids (MUFAs), polyunsaturated fatty acids (PUFAs), unsaturated fatty acids (UFAs), the MUFAs/SFAs ratio, PUFAs/SFAs ratio, atherogenic index (AI), thrombogenic index (TI), the hypocholesterolemic to hypercholesterolemic ratio (h/H), health-promoting index (HPI), hypocholesterolemic index (HI), unsaturation index (UI), saturation index (SI), peroxidizability index (PI), nutritional value index (NVI), hypocholesterolemic index of fatty acids (DFAs), hypercholesterolemic index of fatty acids (OFAs), and the DFAs/OFAs ratio. These indices were calculated based on their fatty acid composition to provide comprehensive nutritional information. A health risk assessment revealed the safety and minimum health risk for the population from consuming the investigated pork cuts using the Target Hazard Quotient (THQ), Hazard Index (HI), and target cancer risk (TR). The ANOVA test showed significant differences in the levels of K, Fe, Mn, Zn, MUFAs, and AI among the pork cut samples. It was noted that by employing the correlation between the fatty acids profile, nutritional indices, and elemental concentrations and an unsupervised statistical method, such as PCA, a perfect separation from the different pork cuts could not be obtained.

## 1. Introduction

Pork ranks among the most extensively produced and consumed meat types globally, serving as a vital source of animal protein for humans because of its nutritional value, distinctive chemical composition, and well-balanced protein content [1]. Lately, nutritional regulation has emerged as a viable and safe approach for improving pork quality [2].

The assessment of meat quality is of paramount importance in ensuring consumer health and satisfaction. One significant aspect of meat quality that has garnered attention in recent years is its nutritional composition. Understanding the nutritional characteristics of meat is vital not only for consumers who seek to make informed dietary choices but also for producers and policymakers aiming to enhance food quality standards [3].

The combined analysis of fatty acids and mineral elements in pork meat presents a comprehensive picture of its nutritional composition and potential health implications. Fatty acids, such as saturated (SFAs), monounsaturated (MUFAs), and polyunsaturated (PUFAs), influence the meat’s nutritional quality. Ratios like MUFAs/SFAs and PUFAs/SFAs provide insights into their impact on cholesterol levels and cardiovascular health. Nutritional indices, including AI, TI, h/H, HPI, HI, UI, SI, PI, NVI, DFAs, OFAs, and DFAs/OFAs, quantify the overall nutritional quality. These indices provide insights into how pork meat might impact inflammation, oxidative stress, and lipid metabolism. Mineral elements like potassium (K), iron (Fe), zinc (Zn), and copper (Cu) contribute to various bodily functions. Balancing these minerals is crucial for optimal health, but an excessive intake of elements like lead (Pb) or cadmium (Cd) can pose risks.

The concern regarding the hazard linked to the presence of toxic and potentially toxic elements in food items, particularly in meat, represents a significant issue in terms of food safety and poses a substantial threat to human health [4]. 

In this context, *one aim* of the present study was to determine the fatty acids (C4:0, C8:0, C10:0, C12:0, C14:0, C15:0, C16:0, C17:0, C18:0, C20:0, C21:0, C22:0, C23:0, C14:1, C16:1, C17:1, C18:1n9, C18:2n6, C18:3n6, C18:3n3, C20:2, C20:3n6, C20:3n3, C20:4n6) and elemental profiles (Na, Mg, Ca, K, Zn, Cu, Mn, Fe, Cr, Li, Co, Mo, V, As, Cd, Sn, Sb, Tl, Ni, Pb, In) of 53 pork cut samples (leg, loin, and tenderloin). Eighteen nutritional indices (SFAs, MUFAs, PUFAs, UFAs, MUFAs/SFAs, PUFAs/SFAs, AI, TI, h/H, HPI, HI, UI, SI, PI, NVI, DFAs, OFAs, and DFAs/OFAs) were employed. *The second objective* was to evaluate the health risks to humans from consuming pork meat in relation to the intake of potentially toxic elements (As, Cd, Pb, Cu, and Cr). To find the significant differences in fatty acid contents and the elemental concentration among pork meat cuts (leg, loin, and tenderloin), a one-way analysis of variance (ANOVA) was used. In addition, principal component analysis (PCA) was applied to establish the possible correlations between the analyzed parameters and different pork cuts. 

## 2. Materials and Methods

### 2.1. Samples Description

Research was conducted on a total set of 53 pork meat samples. The samples consisted of three cuts of pork meat: the leg (*Musculus femoral*) (*n* = 24), loin (*Musculus longissimus dorsi*) (*n* = 18), tenderloin (this element consists of the following muscles: *Musculus psoas major* or *Musculus iliacus*, which may be part of this zone depending on how the meat is cut) (*n* = 11). These samples were procured from local producers and supermarkets in Transylvania, Romania. 

### 2.2. Fatty Acid Profile Analysis

The analysis of the fatty acid profile of pork meat was conducted using gas chromatography with flame ionization detection (GC-FID). In this study, fat extraction from the pork meat samples was carried out using a method developed by our research group. The extraction involved a solution of 0.5 M of sodium hydroxide (NaOH)/methanol (CH_3_OH) at 100 °C for 1 h. Subsequently, fat methylation was performed using 3 M of HCl/CH_3_OH at 100 °C for 30 min. The resulting fatty acid esters were then extracted using n-hexane. This method ensures a comprehensive and accurate analysis of the fatty acid composition in pork meat. The chromatographic separation of fatty acid methyl esters was performed using a gas chromatograph (Trace GC Ultra from Thermo Electron Corporation (Milan, Italy)), using a DB-FATWAX column, 30 m × 0.25 mm × 0.25 µm (Agilent Technologies (Santa Clara, CA, USA)). The analysis was carried out at an injector and a detector temperature of 250 °C, with the column temperature following the oven time–temperature programmer as follows: initiation at 50 °C (maintained for 2 min), programmed at 4 °C/min from 50 °C to 220 °C (held isothermally at 220 °C for 20 min), sampled at a split ratio of 10:1, and utilizing helium as the carrier gas. Each analysis had a total run time of 64.5 min. Fatty acids were identified based on their relative retention time using Supelco^TM^ 37 Component FAME Mix formulas (Sigma-Aldrich Co., LLC, St. Louis, MO, USA.

### 2.3. Calculation of Nutritional Quality Indices (NQIs)

Fatty acid composition data were used to calculate the nutritional indices, which served as indicators for the evaluation of pork meat quality, as follows:

(1) SFA=∑C4:0+C6:0+C8:0+C10:0+C12:0+C13:0+C14:0+C15:0+C16:0+C17:0+C18:0+C20:0+C21:0+C22:0+C23:0+C24:0,                                               

(2) MUFA=∑C14:1+C15:1+C16:1+C17:1+C18:1n9t+C18:1n9c+C20:1n9+C22:1n9+C24:1n9,

(3) PUFA=∑C18:2n6t+C18:2n6c+C18:3n6+C18:3n3+C20:4n6+C20:5n3+C22:2+C22:6n3,

(4) UFA=∑MUFA+PUFA,

The ratios of *MUFAs*/*SFAs* and *PUFAs*/*SFAs* were calculated from the values obtained with Equations (1)–(3).

The AI and TI indices were determined on the fatty acid composition and were computed using the following formulas [5]:

(5) $$AI=\frac{C12:0+\left(4\times C14:0\right)+C16:0}{\sum MUFA+\sum (n-6)PUFA+\sum (n-3)PUFA},$$

(6) $$TI=\frac{C14:0+C16:0+C18:0}{\left(0.5\times \sum MUFA\right)+\left(0.5\times \sum (n-6)PUFA\right)+\left(3\times \sum (n-3)PUFA\right)+\left(\sum (n-3)PUFA/\sum (n-6)PUFA\right)},$$

The *h*/*H* ratio, which represents the relationship between hypocholesterolemic and hypercholesterolemic factors, was determined using the model previously developed in another study [6].

(7) $$h/H=\frac{\sum C18:1n9+C18:2n6+C18:3n3+C20:5n3+C22:5n3+C22:6n3}{\sum C14:0+C16:0},$$

In 2004, a study reported by USA researchers [7], introduced the concept of the health-promoting index (HPI) to evaluate the nutritional quality of dietary fat, with a particular emphasis on its impact on cardiovascular health. The formula for this index is as follows:

(8) $$HPI=\frac{\sum UFA}{C12:0+\left(4\times C14:0\right)+C16:0},$$

The calculation of the HI followed this equation [8]:

(9) $$HI=\frac{\sum C18:1+C18:2+C18:3+C20:3+C20:4+C20:5+C22:4+C22:6}{\sum C14:0+C16:0},$$

The unsaturation index (*UI*) gauges the degree of unsaturation in lipids by calculating the sum of the percentages of each unsaturated fatty acid, multiplied by the number of double bonds present within that specific fatty acid using formula [9]:

(10) $$UI=1\times \left(\%\;monoenoics\right)+2\times \left(\%\;dienoics\right)+3\times \left(\%\;trienoics\right)+4\times \left(\%\;tetraenoics\right)\;+5\times \left(\%\;pentaenoics\right)+6\times \left(\%\;hexaenoics\right),$$

The calculation of the *SI* was performed utilizing the formula recommended by a previous study [5]:

(11) $$SI=\frac{C14:0+C16:0+C18:0}{MUFA+PUFA},$$

The *PI* was determined using the formula suggested by a previous study [10]:

(12) $$PI=0.025\times \left(\%\;monoenoics\right)+1\times \left(\%\;dienoics\right)+2\times \left(\%\;trienoics\right)+4\times \left(\%\;tetraenoics\right)\;+6\times \left(\%\;pentaenoics\right)+8\times \left(\%\;hexaenoics\right),$$

The *NVI* ratio of fatty acids indicates the correlation between saturated fatty acids (C12:0, C14:0, C16:0) and the combined content of oleic and linoleic acids [11].

(13) $$NVI=\frac{C12:0+C14:0+C16:0}{C18:1n9+C18:2n6},$$

The formulas provided below were utilized for computing the *DFAs* and *OFAs* [12,13]:

(14) DFA=UFA+C18:0,

(15) OFA=C12:0+C14:0+C16:0,

The ratio of *DFAs*/*OFAs* was calculated from the values obtained with Equations (14) and (15).

### 2.4. Elemental Profile Analysis

For elemental analysis (Mg, Ca, Na, K, Cu, Fe, Mn, Zn, Cr, Co, Mo, Li, V, As, Cd, Sb, Ni, Tl, Sn, In, and Pb), the ICP-MS technique, using an Elan DRC(e), Perkin Elmer SCIEX^®^, (Wellesley, MA, USA), mass spectrometer was used. The operational parameters comprised a nebulizer gas flow at 0.92 L/min, auxiliary gas flow at 1.2 L/min, plasma gas flow at 15 L/min, a lens voltage of 7.25 V, radiofrequency power set at 1100 W, CeO/Ce ratio of 0.025, and Ba^++^/Ba^+^ ratio of 0.020. Before conducting the analysis, the pork meat samples underwent digestion using a combination of nitric acid (HNO_3_) and hydrogen peroxide (H_2_O_2_) in a microwave system (Speed ENTRY by Berghof^®^, Berlin, Germany). In summary, 500 mg of each sample (fresh weight) was placed into a PTFE digestion vessel. Following this, 7 mL of 60% *v*/*v* HNO_3_ and 1 mL of 30% *v*/*v* H_2_O_2_ were added. The microwave system was programmed to gradually increase the temperature from room temperature to 50 °C in 2 min and maintain this temperature for 5 min. It then increased to 75 °C over 2 min and sustained this temperature for 15 min. Subsequently, it rose to 190 °C in 5 min and held this temperature for 20 min before decreasing to 75 °C over 5 min and maintaining this for an additional 10 min. The digested solutions were allowed to cool to room temperature and then diluted with ultrapure water from a Millipore water purification system, reaching a final volume of 50 mL. Standard stock solutions were prepared using certified multi-element solutions of 10 µg/mL (Ag, Al, As, Ba, Be, Bi, Ca, Cd, Co, Cr, Cs, Cu, Fe, Ga, In, K, Li, Mg, Mn, Na, Ni, Pb, Rb, Se, Sr, Tl, U, V, and Zn) and 10 mg/L (Au, Hf, Ir, Pd, Pt, Rh, Ru, Sb, Sn, and Te) obtained from PerkinElmer Pure Plus in Billerica, MA, USA, dissolved in ultrapure water. Working solutions for the calibration curve were created by diluting specific concentrations and volumes of the stock solution. The concentration range was between 0.1 and 0.5 mg/L for Mg, Ca, Na, and K; 0.01 and 25 µg/L for Zn, Cu, Cr, Mn, Fe, and Ni; 0.005 and 0.5 µg/L for Li, V, Co, As, Mo, Cd, In, Sn, Sb, Tl, and Pb, respectively. The method’s accuracy was checked using NCS ZC85006 and NCS ZC73016 as standard reference materials. 

### 2.5. Risk Assessment of Toxic and Potentially Toxic Element Contamination in Pork Meat

Health risk assessments play a crucial role in ensuring the safety of pork meat by identifying and evaluating potential hazards that could pose risks to consumers. In this context, following the guidelines established by the United States Environmental Protection Agency (US EPA) [14,15], an evaluation of non-cancerous risks linked to the consumption of various pork cuts containing Arsenic (As), Cadmium (Cd), Lead (Pb), Copper (Cu) and Chromium (Cr) among the population was carried out. This assessment relied on computed values such as the Target Hazard Quotient (*THQ*) and Hazard Index (*HI*), calculated using the formulas detailed in references [16,17]:

(16) $$Exposure\;dose=\frac{C_{i}\times D_{i}\times E_{d}}{Bw\times A_{t}},$$

(17) $$Tageted\;Hazard\;Quotient\;\left(THQ\right)=\frac{Exposure\;dose}{R_{f}D},$$

(18) $$Hazard\;Index\;\left(HI\right)=\sum_{k=1}^{n=k}Targeted\;Hazard\;Quotient,$$

where *C_i_* represents the average concentration of the metal in pork cuts (measured in mg/kg wet weight), *D_i_* signifies the daily intake of meat (100 g/person/day), *E_d_* indicates the average exposure duration (70 years), *B_w_* denotes the average weight (70 kg), *A_t_* represents the average lifetime (70 years) and *R_f_D* stands for the recommended reference dose (As (0.3 µg/kg bw/day), Cd (1 µg/kg bw/day), Pb (3.5 µg/kg bw/day), Cu (40 µg/kg bw/day) and Cr (3 µg/kg bw/day) [18]. *HI* was computed by summing the Target Hazard Quotients (*THQs*) for As, Cd, Pb, Cu, and Cr because individuals experience combined effects from exposure to multiple contaminants [19]. If the calculated HI is less than 1, it implies that the exposed population is presumed to be safe [20].

Furthermore, to assess the potential carcinogenic risk posed by metals present in meat samples, the target cancer risk was determined utilizing the next equation [21]:

(19) $$TR=\frac{E_{d}\times EF\times {IR}_{d}\times C\times {CPS}_{o}}{B_{w}\times A_{t}}\times 10^{-3},$$

where *TR* represents the target cancer risk; *CPS_o_* is the carcinogenic potency slope, oral (mg/kg Bw/day); *B_W_* is the average body weight (70 kg); and *A_t_* represents the average lifetime (70 years). The *CPS_o_* values are 1.5 for As, 0.38 for Cd, 0.0085 for Pb, 1.5 for Cu, and 0.5 for Cr, respectively [21].

### 2.6. Chemometric Analysis

All chemometric methods were carried out using SPSS v.20 (IBM, New York, NY, USA) software. To identify significant differences between pork cuts (the leg, loin, and tenderloin), a one-way ANOVA was conducted, followed by post hoc Tukey’s test (multiple comparison tests). Any differences associated with *p*-values less than 0.05 were considered to be statistically significant. 

PCA was applied to establish the possible correlations among the analyzed parameters and different pork cuts. PCA is one of the most widely employed unsupervised pattern methods. This technique can divide a large data set into principal components (PCs). In doing so, it minimizes the loss of original information. The analysis effectively eliminates multicollinearity among features and consolidates highly correlated variables into a set of uncorrelated variables (PCs). These obtained PCs are presented in descending order of importance, accompanied by their eigenvalues—a crucial measure of a component’s significance in contributing to the variance of the data set. Typically, the first two or three components retain a substantial percentage of the data variance. In the present study, PCA was applied for a reduction in the obtained experimental data matrix after GC-FID and ICP-MS analysis. 

### 2.7. Ethical Statement

Ethical review and approval were not required for this study as it involved research on a commercially available food product: pork meat cuts.

## 3. Results and Discussion

### 3.1. Fatty Acid Composition and Nutritional Indices

The experimental analysis of the fatty acid profile in different pork cut samples (the leg, loin, and tenderloin) was conducted using GC-FID, providing valuable insights into the composition of fatty acids present in the meat. The analysis revealed a diverse array of fatty acids present in pork meat. The outcome of our analysis pointed out that among the 24 fatty acids identified, the levels of 5 fatty acids significantly differed among the leg, loin, and tenderloin of pork meat. Table 1 displays the percentages of various fatty acids in pork leg, loin, and tenderloin, representing their composition in relation to total fatty acids. The table also provides the percentages of SFAs, MUFAs, PUFAs, and UFAs, the relevant fatty acid ratios of meat samples, and *p*-values obtained from the ANOVA test.

The meat’s characteristics are influenced by both the quantity of fat and its composition, particularly the ratio of individual fatty acids. Fatty acids play a pivotal role not only in the nutritional content but also in determining the tenderness and shelf life of the meat. The balance between SFAs, MUFAs, and PUFAs in pork meat is a critical factor influencing both its nutritional quality and sensory characteristics. SFAs, while contributing to the meat’s texture, may also be associated with potential health concerns when consumed in excess. MUFAs, on the other hand, contribute to tenderness and flavor. The presence of PUFAs, particularly omega-3 and omega-6 fatty acids, has important nutritional implications, as they are essential for human health. The distribution of individual fatty acids in pork typically adheres to the following sequence: MUFAs > SFAs > PUFAs [22]. Oleic acid (C18:1n9) (averaging 29–39%) is the predominant fatty acid among MUFAs, while palmitic acid (C16:0) (averaging 22–24%) and stearic acid (C18:0) (averaging 20–25%) usually dominate among SFAs. Among PUFAs, linoleic acid (C18:2n6) typically constitutes the largest share, accounting for an average of 13–17% of total fatty acids [22].

Among the different cuts of pork, tenderloin stands out with the highest concentration of SFAs, accounting for 50.70% of its lipid composition. In contrast, the lipid content in the leg and loin contains a relatively lower proportion of SFAs, with approximately the same amount of fatty acids at 44.57% and 45.76%, respectively. The concentration of C14:0 ranged from 0.37% to 1.90% in the leg, from 0.42% to 1.48% in the loin, and from 0.48% to 4.67% in the pork tenderloin. C16:0 showed variations between 14.56% and 29.81% in the leg, between 19.33% and 27.93% in the loin, and between 19.30% and 29.22% in the pork tenderloin. As for C18:0, its concentration ranged from 8.43% to 34.43% in the leg, from 8.88% to 35.96% in the loin, and from 13.94% to 37.32% in the pork tenderloin. It is worth noting that the highest quantities contributing to a higher total of SFAs in the pork tenderloin were the C16:0 and C18:0.

Following SFAs, among the most abundant fatty acids in pork meat, MUFAs take the second spot in the lipid composition. Oleic acid (C18:1n9) was the most predominant fatty acid in the pork leg (39.00 ± 0.71), pork loin (37.93 ± 0.65), and pork tenderloin, respectively (29.89 ± 0.52). The MUFA content in the leg and loin was notably higher, with levels of 40.53% and 39.18%, respectively, and nearly 10% lower at 31.09% in the pork tenderloin. The most common monounsaturated fatty acid identified in pork meat was C18:1n9. These were also confirmed by the ANOVA test, which revealed differences in the MUFA content between the various cuts of pork meat. MUFA levels in the pork leg were significantly higher than in the loin and tenderloin (*p* = 0.048).

PUFAs include linoleic (C18:2n6), alpha-linolenic (C18:3n3), and *cis*-11,14-eicosadienoic (C20:2) fatty acids. Particularly, the pork tenderloin exhibited higher contents of C18:2n6 (16.97% ± 0.81) and C18:3n3 (0.73 ± 0.09) than the pork leg and loin. The tenderloin contained the highest concentration of PUFAs at 18.20%, with a slightly lower amount in the leg and loin’s lipid fractions (14.92% and 15.06%, respectively), which was nearly 3% less.

The interplay of SFAs, MUFAs, and PUFAs in pork meat plays a pivotal role in shaping its nutritional value and sensory attributes. Striking the right balance is imperative to meet consumer preferences for flavor, tenderness, and overall enjoyability, all while adhering to health-focused dietary recommendations [23]. 

In recent years, there has been growing concern about the health risks associated with the consumption of pork fat, which has been linked to various diseases, including cancer, cardiovascular conditions, and diabetes [24]. The evaluation of the significance of fat content in meat has evolved over time. Presently, advancements in our ability to meticulously analyze individual fatty acids and their impact on tissue metabolism have led to a nuanced understanding of the role of fats in our diet. According to the recommendations of the Food and Agriculture Organization of the United Nations [25], the ideal ratio of unsaturated to saturated fatty acids in the diet should be 1:1. In the examined meat samples, this ratio decreased in the following order: leg (1.245) > loin (1.195) > tenderloin (0.972). 

Therefore, in nutritional assessments, the PUFAs/SFAs ratio is commonly employed to gauge the nutritional quality of pork fat [26]. Essentially, a higher PUFAs/SFAs ratio signifies greater nutritional value in the meat. The pork tenderloin had a higher C16:0 level (23.92 ± 0.73), which resulted in a significantly higher content of total SFAs (50.70%) in this pork cut type compared to the pork leg and loin (44.54 and 45.76%). Interestingly, the pork tenderloin exhibited higher total levels of UFAs (49.29%), especially PUFAs (18.20%). Furthermore, the pork tenderloin had greater levels of total n-6 fatty acids due to its higher levels of C18:2n6 and C20:3n6, which led to a significantly higher n-6/n-3 fatty acids ratio (0.159) as well as PUFAs/SFAs ratio (0.0367) compared to the pork leg and loin. In the studies previously carried out [27,28], they established that the composition of fatty acids in meat is highly influenced by slaughter weight, environmental and nutritional factors, and from the previous research carried out [29], another factor found to influence the fatty acid composition is the metabolism of lipid stores during sow lactation. Also, in a study conducted by researchers from the UK [30], it was reported that the proportion of energy available for fat deposition in pigs increased during growth, leading to an increased rate of de novo fatty acid synthesis. Therefore, the differences in the fatty acid composition between the pork leg, loin, and tenderloin could be due to the above-mentioned factors, and these results also suggest that the de novo synthesis of UFAs was greater in the pork tenderloin than in the pork leg and loin. Furthermore, when compared with the fatty acid compositions of pork meat cuts in the study conducted by researchers from Poland [31], a lower total SFA content was reported (43.81%) but with a higher UFA level (56.18%) and PUFAs (16.195) in the *longissimus lumborum* muscle of finisher pigs. Apart from being the major energy source in the human body, fatty acids are important for many biological processes, especially n-3 PUFAs, which produce a lot of beneficial effects on health, such as reducing the risk of heart disease and stroke reductions [32]. Furthermore, n-3 PUFAs prevent cancer, diabetes, and other inflammatory and autoimmune diseases. In the present study, the levels of n-3 fatty acids such as C18:3n-3 and C20:3n3 were higher in pork tenderloin meat compared to the pork leg and loin. According to the nutritional recommendations, the n-6/n-3 PUFAs ratio in the human diet should not exceed 4.0 because a higher ratio is associated with an increased risk of cancer, while the PUFAs/SFAs ratio should be above 0.45 [33]. According to the outcome of our analysis, all cuts of pork meat exhibited an n-6/n-3 ratio under the recommended value of less than 4.0, while the value of the PUFAs/SFAs ratio was under the recommended value of 0.45 for the meat (pork leg (0.355), loin (0.330) and tenderloin (0.374)), which was higher than the value (0.36) reported for the tenderloin muscle of finisher pigs in the study conducted by researchers from Poland [31] and lower than the values (0.36 to 0.53) reported for the *longissimus dorsi muscle* of finisher pigs in the study conducted by researchers from Spain [34]. Thus, it may be said that the meat of pork loin partly shows a “healthier” fatty acids profile as it possesses a higher total UFA content and a more favorable PUFAs/SFAs ratio than the meat of the pork leg and tenderloin.

To assess the potential health effects of different cuts of pork meat, various nutritional indices, including the TI, AI, h/H, HPI, HI, UI, SI, PI, NVI, DFAs, OFAs, and DFA/OFAs were computed based on the fatty acid composition (Table 2). The results were determined by computing the mean ± SD. *p*-Values from the ANOVA test, which are also indicated in Table 2. 

The AI of food quantifies its capacity to potentially contribute to the onset of atherosclerosis; this is a medical condition marked by the accumulation of plaque within the arteries. This index is derived from the ratio of distinct fatty acids, particularly the proportion of SFAs to UFAs [35]. A lower AI value signifies a reduced proportion of saturated to unsaturated acids, which, in turn, diminishes the endothelial attachment of lipids and plaque formation within blood vessels. Similarly, the TI is computed from the proportion of other fatty acids, with a lower index indicating a reduced risk of blood coagulation disturbances and clot formation [36]. These indices (AI and TI) play a crucial role in discerning the nutritional advantages offered by the lipids in pork meat [37]. Pork meat, much like various other animal meat, comprises a blend of fatty acids, encompassing both SFAs and UFAs. The AI of pork meat is subject to fluctuations due to factors such as the specific meat cut, the cooking techniques employed, and the dietary habits of the animal. Typically, pork is recognized for containing a greater ratio of SFAs compared to certain alternative meats like poultry or fish [38]. In our study, both indices showed a favorable low level, closely mirroring the values observed in the leg and loin, along with an increase in tenderloin meat. These are also confirmed by the ANOVA test, which revealed differences in the AI of different cuts of pork meat. The AI in the pork tenderloin was significantly higher than leg and loin (*p* = 0.036). 

The results showed that the HPI, HI, and UI values were notably higher in the pork leg compared to the other pork cuts. The DFA values for the other pork cuts were similar, while the SI, PI, NVI, OFA, and DFA/OFA values for the leg and loin exceeded those for tenderloin meat. Additionally, the h/H values for the leg and loin were higher than those for tenderloin, which had the lowest h/H value. Consequently, the elevated AI, NVI, and TI values observed in the pork leg suggest that the consumption of fatty acids from this pork cut may pose a higher health risk compared to the other cuts of pork [8]. 

### 3.2. Elemental Profile and Risk Assessment of Selected Pork Meat Cuts Based on Heavy Metal Content

The concentration of twenty-one elements classified based on their concentration levels in different pork cut samples (the leg, loin, and tenderloin) are reported as the mean ± standard deviation on a fresh weight basis, and *p*-values from the ANOVA test are indicated in Table 3. In the present study, the concentrations of macroelements (Na, Mg, Ca, and K), microelements (Zn, Cu, Mn, Fe, and Cr), and trace elements, namely, Li, Co, Mo, and V (considered to be non-toxic) were assessed in pork meat. Besides these elements, non-essential elements with toxic potential, such as As, Cd, Sn, Sb, Tl, Ni, Pb, and In, were analyzed in the current study.

The dominant mineral was K (3389.97–4617.76 mg/kg for the *leg*; 3948.85–9045.76 mg/kg for loin and 3718.54–5779.58 mg/kg for tenderloin), followed by Na (362.42–2369.41 mg/kg for the leg; 318.66–922.41 mg/kg for loin and 387.64–689.82 mg/kg for tenderloin), Mg (147.91–317.24 mg/kg for the *leg*; 192.94–709.64 mg/kg for loin and 228.52–302.75 mg/kg for tenderloin) and Ca (13.13–247.44 mg/kg for the leg; 5.29–623.58 mg/kg for loin and 12.19–45.09 mg/kg for tenderloin). This finding regarding the descending order of macro minerals agrees with previous results obtained by researchers from Serbia [39], Romania [40], China [41], and Korea [42]. 

Macro elements are crucial for numerous physiological functions within the body, including blood clotting [43], osmotic pressure regulation [44], maintaining an acid-base balance [45], facilitating muscle contractions [43], promoting bone development [43], supporting enzymatic activities [46], and aiding in hemoglobin synthesis [47]. The ratio between Na and K in any dietary item is of significant importance. Elevated sodium intake coupled with low potassium intake can contribute to a higher prevalence of hypertension, which is a condition characterized by elevated blood pressure [48]. Various studies have highlighted the substantial impact of the Na:K ratio on both hypertension prevalence and blood pressure regulation [49,50,51]. Maintaining a balanced Na-to-K ratio is crucial in preventing diet-induced secondary hypertension, which is a recognized risk factor for cardiovascular disease [51]. Ideally, the Na/K ratio within our body should be less than one [52]. In this current study, the Na/K ratio observed in pork meat was less than one, with values of 0.145 (for the leg), 0.109 (for loin), and 0.111 (for tenderloin), respectively. These findings suggest that the consumption of examined pork meat varieties could potentially benefit human health and might contribute to managing high blood pressure.

Zn, Cu, Mn, Fe, and Cr displayed mean values of more than 0.1 mg/kg in selected pork cut samples, and all five were taken as minor elements in the current study. As shown in Table 3, the highest mean values of Fe were found in the leg and tenderloin samples, while Zn was the mineral with the highest mean level in loin samples. The mean levels of these essential elements were established within the following ranges: Fe (2.71–30.66 mg/kg, in the leg; 3.07–14.64 mg/kg, in loin and 7.20–25.59 mg/kg, in tenderloin); Zn (4.35–25.94 mg/kg, in the leg; 5.00–11.04 mg/kg, in loin and 6.59–23.31 mg/kg, in tenderloin); Cu (0.01–2.20 mg/kg, in the leg; 0.12–1.53 mg/kg, in loin and 0.35–2.91 mg/kg, in tenderloin); Cr (0.04–1.42 mg/kg, in the leg; 0.21–1.08 mg/kg, in loin and 0.20–1.07 mg/kg, in tenderloin), and Mn (0.05–0.64 mg/kg, in leg; 0.04–0.18 mg/kg, in loin and 0.09–0.29 mg/kg, in tenderloin).

The obtained results for trace elements showed that the levels in loin and tenderloin samples were highest for Li (0.03 mg/kg, 0.04 mg/kg) and V (0.05 mg/kg, 0.04 mg/kg), followed by Mo (0.02 mg/kg) and Co (0.004 mg/kg). For leg samples, the mean concentrations were 0.04 mg/kg for Li, 0.05 mg/kg for V, 0.03 mg/kg for Mo, and 0.01 mg/kg for Co.

Toxic and potentially toxic elements, such as As, Cd, Pb, Sn, etc., can be present in food, and their concentrations can vary depending on various factors (e.g., environmental conditions, production methods, food processing, etc.). These elements are toxic due to their harmful effects on humans when their levels exceed the regulated limit [53]. The mean concentrations of potentially toxic elements, namely Ni, Pb, As, In, Sb, Cd, and Tl, in investigated meat samples are indicated in Table 3. Most research on pork looks at the average level of elements without saying which muscle was tested. Studies from Iran [20], Korea [42], and China [17] show varied levels of elements like Mg, Cu, Fe, Mn, Zn, and Mo in pork meat. For example, Iran found Mg levels between 355 and 1266 mg/kg and Cu between 1.8 and 7.2 mg/kg, Korea reported K between 3600 and 4430 mg/kg and Na between 360 and 410 mg/kg, and China noted K levels at 15,046 and 14,369 mg/kg. Research from Croatia and Serbia [54,55] gives a wide range for Mg, Ca, K, Zn, and Fe, as follows: Mg (17–346 mg/kg in one study and 182–258 mg/kg in another); Ca (0.36 to 65 mg/kg); K (2.6 to 4440 mg/kg); Zn (12.1 to 30.4 mg/kg); and Fe (0.67–56 mg/kg and 3.40–8.88 mg/kg). A South African study [56] looked at two types of pork cuts and listed their mineral content in mg/100 g, with loin and leg cuts showing different levels of Mg (23.78), Ca (28.5), K (286.8), Na (71.2), Fe (0.17), and Zn (0.20).

The statistical analysis, conducted using the ANOVA test, revealed notable differences in the levels of K, Fe, Mn, and Zn among the various pork cuts. In the present study, the concentration of K in pork loin was significantly higher than in the pork leg (*p* = 0.044). Also, significant differences between leg and loin samples were found for Fe, Mn, and Zn (*p* = 0.002 for Fe, *p* = 0.015 for Mn, and *p* = 0.029 for Zn, respectively).

From the obtained results, it can be observed that there are variations in the concentrations of the investigated elements in selected pork meat cuts for our study. Several factors can contribute to these variations, including the cut of meat [57,58], the animal’s diet [57,58], the animal’s age and breed [29], and the geographic location and environment in which pigs are raised.

Health risk assessments play a crucial role in ensuring the safety of pork meat by identifying and evaluating potential hazards that could pose risks to consumers. In this context, the average concentrations of five elements, such as As, Cd, Pb, Cu and Cr, were utilized to evaluate the potential health risks associated with consuming various pork cuts (leg, loin, and tenderloin). THQ, HI, and TR were used to estimate health risks according to USEPA guidelines [14]. The safe values for daily intake (µg/kg bw/day) are established as 3.0 for As, 0.8 for Cd, 1.5 for Pb, 40 for Cu, and 3000 for Cr. As shown in Table 4, the mean exposure doses (µg/kg bw/day) of As, Cd, Pb, Cu, and Cr by different pork meat cuts consumption were 0.041, 0.011, 0.097, 1.878 and 0.914 (leg), 0.024, 0.003, 0.048, 1.691 and 0.880 (loin) and 0.024, 0.006, 0.072, 2.640 and 0.880 (tenderloin), which is far below the safe values. As for the non-carcinogenic risk linked to metals, parameters such as THQ and HI were computed, and their values are detailed in Table 4. TR is a tool utilized specifically in health risk assessment to evaluate the potential cancer risk associated with exposure to certain analyzed metals. The risks of 10^−6^, 10^−5^, or 10^−4^ signify contaminant concentrations that, over a lifetime of exposure, could result in one cancer case in a population of one million, 100,000, or 10,000 people, respectively [59]. The TR values corresponding to this assessment are presented in Table 4. 

THQ and HI results were lower than one, suggesting a minimal risk of non-carcinogenic consequences per metal for consumers who eat the investigated pork meat samples. TR values were within the guideline value, which implies that none of the analyzed metals in the present study pose a carcinogenic risk.

### 3.3. Chemometric Processing

**Figure 1:**
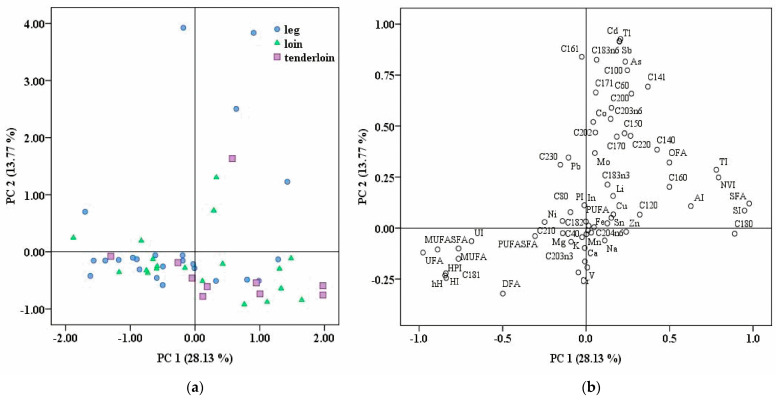
(**a**) Score plot of pork cuts investigated (leg, loin, and tenderloin) using the first two principal components (PC1 and PC2); (**b**) loading plot of analyzed variables obtained using PC1 and PC2, explaining a total variance of 41.9%.

An unsupervised PCA model was constructed to assess if samples from different pork cuts offer the potential to cluster into separate groups based on their fatty acids profile, nutritional index, and elemental concentrations. In this study, the first 13 PCs had eigenvalues higher than one and explained a cumulative variance of 89.28%. The score plot of pork cuts and the loading plot using the first two principal components are indicated in Figure 1a and 1b, respectively. It can be observed that using the association between fatty acid profiles, nutritional indices, elemental concentrations, and the unsupervised statistical method, a perfect separation could not be obtained. The levels of C18:0, SFAs, UFAs, MUFAs/SFAs, h/H, HPI, HI, UI, and SI were the most influential factors for PC1 in the pork meat samples, explaining 28.13% of the variability. PC2 (C18:3n6, C16:1, C10:0, Tl, Sb, and Cd) explained an additional 13.77% of the variability. The third principal component, PC3, contributed to 11.19% of the variance based on C18:2, C18:3n3, PUFA, PUFA/SFA, PI, and DFA/OFA. In addition, other principal components, namely PC4 (C21:0, C8:0, and C4:0), PC5 (C12:0), and PC6 (Mg and K) explained, in total, an additional percentage of 22.47% of the variability.

## 4. Conclusions

In the present study, the fatty acids and elemental profiles of 53 pork cut samples (leg, loin, and tenderloin) were assessed. To offer valuable insights into their potential health implications, 18 nutritional indices were evaluated. Also, this study provides information on the levels of various metals (As, Cd, Pb, Cu, and Cr) in pork meat from Romania and calculates the potential health risk toxicity associated with their consumption.

This study reveals that fatty acid compositions vary across different pork cuts, with the tenderloin showing the highest SFA levels. These variations affect meat’s quality, flavor, and healthfulness, highlighting the need for a balanced intake of SFAs, MUFAs, and PUFAs. In addition, nutritional indices suggest that leg and loin cuts may be healthier choices over tenderloin, offering lower risks for atherosclerosis and thrombosis due to their favorable AI and TI values. This underscores the critical role of choosing specific pork cuts to align with health goals, particularly for those looking to reduce their atherosclerosis risk or improve dietary fat quality through a better PUFAs/SFAs ratio.

The contents of macro minerals (K, Na, Mg, and Ca), micro essential elements (Fe, Zn, Cu, Cr, and Mn), and toxic and potentially toxic elements (Ni, Sn, Pb, as, In, Sb, Cd, and Tl) obtained in this study showed the following order: K > Na > M g> Ca > Fe > Zn > Cu > Cr > Mn > Ni > Sn > Pb > As > In > Sb > Cd > Tl. A comprehensive assessment of human health risks associated with consuming various cuts of pork was conducted, considering concentrations of As, Cd, Pb, Cu, and Cr. The calculated THQ and HI values were significantly lower than one, indicating minimal non-carcinogenic risks associated with each metal for individuals consuming the tested pork samples. Furthermore, the TR values fell within the recommended guidelines, suggesting that none of the metals analyzed in this study present a carcinogenic risk. 

The ANOVA test revealed significant differences in K, Fe, Mn, Zn, MUFA levels, and AI among the pork cuts. PCA was performed to assess if samples from different pork cuts offer the potential to cluster into separate groups based on their fatty acids profile, nutritional index, and elemental concentrations. It could be observed that using the association between the previously mentioned parameters and the unsupervised statistical method (PCA), a perfect separation could not be obtained.

## Figures and Tables

**Table 1:** Fatty acid composition in pork leg, loin, and tenderloin (expressed in % of total fatty acids) and the content (%) of SFAs, MUFAs, PUFAs, and UFAs relevant fatty acids ratios of meat samples and *p*-values.

Fatty Acid	Leg	Loin	Tenderloin	*p*-Values
	Concentration (%, Mean ± SD)			
**C4:0**	0.02 ± 0.01	0.00 ± 0.00	0.00 ± 0.00	0.614
**C8:0**	0.01 ± 0.01	0.00 ± 0.00	0.00 ± 0.00	0.557
**C10:0**	0.04 ± 0.03	0.03 ± 0.01	0.03 ± 0.01	0.881
**C12:0**	0.04 ± 0.03	0.04 ± 0.01	0.16 ± 0.03	0.173
**C14:0**	0.72 ± 0.13	0.71 ± 0.01	1.06 ± 0.01	0.300
**C15:0**	0.00 ± 0.00	0.02 ± 0.01	0.03 ± 0.01	0.518
**C16:0**	22.29 ± 1.76	23.52 ± 1.81	23.92 ± 0.73	0.247
**C17:0**	0.11 ± 0.05	0.11 ± 0.01	0.14 ± 0.01	0.581
**C18:0**	20.49 ± 1.57	20.72 ± 0.01	24.66 ± 0.01	0.241
**C20:0**	0.44 ± 0.08	0.41 ± 0.01	0.44 ± 0.01	0.905
**C21:0**	0.21 ± 0.07	0.14 ± 0.01	0.16 ± 0.01	0.761
**C22:0**	0.07 ± 0.04	0.06 ± 0.01	0.04 ± 0.01	0.394
**C23:0**	0.10 ± 0.05	0.00 ± 0.00	0.05 ± 0.01	0.543
**C14:1**	0.02 ± 0.01	0.00 ± 0.00	0.00 ± 0.00	0.624
**C16:1**	1.40 ± 0.04	1.15 ± 0.08	1.11 ± 0.07	0.267
**C17:1**	0.11 ± 0.07	0.10 ± 0.05	0.09 ± 0.03	0.714
**C18:1n9**	39.00 ± 0.71	37.93 ± 0.65	29.89 ± 0.52	0.057
**C18:2n6**	13.90 ± 0.63	13.97 ± 0.58	16.97 ± 0.81	0.417
**C18:3n6**	0.02 ± 0.01	0.02 ± 0.01	0.01 ± 0.01	0.752
**C18:3n3**	0.52 ± 0.06	0.63 ± 0.08	0.73 ± 0.09	0.519
**C20:2**	0.34 ± 0.05	0.34 ± 0.01	0.33 ± 0.01	0.997
**C20:3n6**	0.10 ± 0.02	0.06 ± 0.03	0.09 ± 0.05	0.609
**C20:3n3**	0.04 ± 0.01	0.04 ± 0.02	0.06 ± 0.03	0.185
**C20:4n6**	0.01 ± 0.01	0.00 ± 0.00	0.00 ± 0.00	0.497
**ƩSFAs**	44.57 ± 8.19	45.76 ± 7.97	50.70 ± 8.62	0.127
**ƩMUFAs**	40.51 ± 9.37	39.18 ± 11.70	31.09 ± 10.45	0.048
**ƩPUFAs**	14.92 ± 7.73	15.06 ± 6.52	18.20 ± 7.68	0.434
**ƩUFAs**	55.43 ± 8.19	54.24 ± 7.97	49.30 ± 8.62	0.127
**MUFAs/SFAs**	0.960 ± 0.347	0.919 ± 0.413	0.652 ± 0.302	0.067
**PUFAs/SFAs**	0.355 ± 0.226	0.330 ± 0.131	0.374 ± 0.188	0.828

**Table 2:** Nutritional indices in pork meat.

Nutritional Index	Leg	Loin	Tenderloin	*p*-Values
**AI**	0.654 ± 0.236	0.723 ± 0.259	1.067 ± 0.834	0.036
**TI**	1.575 ± 0.671	1.584 ± 0.677	1.918 ± 0.729	0.351
**h/H**	2.401 ± 0.699	2.170 ± 0.423	1.948 ± 0.569	0.107
**HPI**	2.312 ± 0.679	2.089 ± 0.417	1.863 ± 0.585	0.105
**HI**	2.427 ± 0.701	2.199 ± 0.420	1.981 ± 0.578	0.115
**UI**	71.022 ± 13.106	70.051 ± 9.065	68.408 ± 12.864	0.832
**SI**	0.827 ± 0.297	0.869 ± 0.291	1.069 ± 0.395	0.115
**PI**	16.541 ± 7.939	16.728 ± 6.975	19.778 ± 7.937	0.477
**NVI**	0.457 ± 0.153	0.480 ± 0.103	0.571 ± 0.230	0.146
**DFA**	75.924 ± 4.031	74.959 ± 2.597	73.961 ± 4.624	0.345
**OFA**	24.076 ± 4.031	25.041 ± 2.597	26.039 ± 4.624	0.345
**DFA/OFA**	14.418 ± 7.623	14.600 ± 6.291	17.706 ± 7.509	0.423

**Table 3:** Average concentration levels of macro, micro, and trace elements in various pork cut samples.

Elements	Leg	Loin	Tenderloin	*p*-Values
	Concentration (mg/kg, Mean ± SD)			
**Mg**	231.80 ± 43.37	276.06 ± 112.29	254.67 ± 21.55	0.156
**Ca**	63.15 ± 57.23	84.25 ± 146.30	26.27 ± 9.28	0.280
**Na**	579.69 ± 398.33	500.12 ± 166.32	481.57 ± 102.67	0.554
**K**	3986.14 ± 319.07	4586.63 ± 1167.50	4333.87 ± 567.67	0.044
**Cu**	1.13 ± 0.70	1.01 ± 0.36	1.58 ± 0.81	0.059
**Fe**	14.28 ± 9.52	6.18 ± 3.11	13.97 ± 6.43	0.002
**Mn**	0.16 ± 0.11	0.09 ± 0.03	0.16 ± 0.04	0.015
**Zn**	11.26 ± 6.32	7.23 ± 1.75	11.35 ± 5.54	0.029
**Cr**	0.55 ± 0.35	0.48 ± 0.24	0.53 ± 0.26	0.746
**Co**	0.01 ± 0.00	0.00 ± 0.00	0.00 ± 0.00	0.292
**Mo**	0.03 ± 0.01	0.02 ± 0.02	0.02 ± 0.01	0.114
**Li**	0.04 ± 0.03	0.03 ± 0.02	0.04 ± 0.02	0.280
**V**	0.05 ± 0.03	0.05 ± 0.02	0.04 ± 0.02	0.933
**As**	0.02 ± 0.02	0.01 ± 0.01	0.01 ± 0.01	0.119
**Cd**	0.01 ± 0.01	0.00 ± 0.00	0.00 ± 0.01	0.253
**Sb**	0.01 ± 0.01	0.00 ± 0.00	0.00 ± 0.01	0.275
**Ni**	0.18 ± 0.10	0.13 ± 0.16	0.17 ± 0.13	0.561
**Tl**	0.01 ± 0.01	0.00 ± 0.00	0.00 ± 0.00	0.246
**Sn**	0.09 ± 0.05	0.08 ± 0.04	0.08 ± 0.04	0.646
**In**	0.01 ± 0.01	0.00 ± 0.01	0.01 ± 0.01	0.269
**Pb**	0.06 ± 0.05	0.03 ± 0.02	0.04 ± 0.04	0.066

**Table 4:** Estimated exposure to As, Cd, Pb, Cu and Cr for population from Romania by consuming different pork meat cuts and their health risk assessment.

Metal	Exposure Dose (µg/kg bw/Day)			THQ			TR		
	Leg	Loin	Tenderloin	Leg	Loin	Tenderloin	Leg	Loin	Tenderloin
**As**	0.041	0.024	0.024	0.137	0.081	0.079	5.2 × 10^−8^	3.1 × 10^−8^	3.0 × 10^−8^
**Cd**	0.011	0.003	0.006	0.011	0.003	0.006	3.7 × 10^−9^	1.1 × 10^−9^	2.0 × 10^−9^
**Pb**	0.097	0.048	0.072	0.024	0.012	0.018	7.1 × 10^−10^	3.5 × 10^−10^	5.2 × 10^−10^
**Cu**	1.878	1.691	2.640	0.376	0.338	0.528	2.4 × 10^−6^	2.1 × 10^−6^	3.3 × 10^−6^
**Cr**	0.914	0.792	0.880	0.305	0.264	0.293	3.9 × 10^−7^	3.3 × 10^−7^	3.7 × 10^−7^
**HI**	0.853	0.699	0.925						

## Data Availability

The original contributions presented in the study are included in the article, further inquiries can be directed to the corresponding author.
